# Recognition of maize seed varieties based on hyperspectral imaging technology and integrated learning algorithms

**DOI:** 10.7717/peerj-cs.1354

**Published:** 2023-05-10

**Authors:** Huan Yang, Cheng Wang, Han Zhang, Ya’nan Zhou, Bin Luo

**Affiliations:** 1 Research Center of Intelligent Equipment, Beijing Academy of Agriculture and Forestry Sciences, Beijing, China; 2 National Agricultural Intelligent Equipment Engineering Technology Research Center, Beijing, China; 3 School of Agricultural Engineering, Jiangsu University, Zhenjiang, China

**Keywords:** Hyperspectral, Random subspace ensemble learning, Maize seed, Variety recognition

## Abstract

Purity is an important factor of maize seed quality that affects yield, and traditional seed purity identification methods are costly or time-consuming. To achieve rapid and accurate detection of the purity of maize seeds, a method for identifying maize seed varieties, using random subspace integrated learning and hyperspectral imaging technology, was proposed. A hyperspectral image of the maize seed endosperm was collected to obtain a spectral image cube with a wavelength range of 400∼1,000 nm. Methods, including Standard Normal Variate (SNV), multiplicative Scatter Correction (MSC), and Savitzky–Golay First Derivative (SG1) were used to preprocess raw spectral data. Iteratively retains informative variables (IRIV) and competitive adaptive reweighted sampling (CARS) were used to reduce the dimensions of the spectral data. A recognition model of maize seed varieties was established using k-nearest neighbor (KNN), support vector machine (SVM), line discrimination analysis (LDA) and decision tree (DT). Among the preprocessing methods, MSC has the best effect. Among the dimensionality reduction methods, IRIV has the best performance. Among the base classifiers, LDA had the highest precision. To improve the precision in identifying maize seed varieties, LDA was used as the base classifier to establish a random subspace ensemble learning (RSEL) model. Using MSC-IRIV-RSEL, precision increased from 0.9333 to 0.9556, and the Kappa coefficient increased from 0.9174 to 0.9457. This study shows that the method based on hyperspectral imaging technology combined with subspace ensemble learning algorithm is a new method for maize seed purity recognition.

## Introduction

Seed purity is an important indicator of seed quality (Wang, Wu & Han, 2021). Seed purity refers to the degree of typical consistency between individual varieties in terms of characteristics. It is expressed by the percentage of the number of seeds of this variety in the number of samples for testing this crop. If the purity of the seeds does not meet the predetermined requirements, there will be an uneven emergence of the maize seeds when the seeds are planted with the field. Plants that are not tall enough will not receive enough sunlight for photosynthesis, which reduces the yield (Jiao et al., 2008) from the maize. According to related studies, if the purity of the maize seeds is agricultural product inspection reduced by 1%, It will lead to a reduction of approximately 2 kg/mus of crop yield. With the widespread application of hybridization technology in the seed industry, the number of varieties of crop seeds is increasing. At the same time, the degree of similarity between different varieties is also increasing. Therefore, it is difficult to distinguish them effectively by relying on human sensory organs. In addition, the phenomena of adulteration and fraud in the seed market occasionally occurs. Harvesting and processing can be prone to confounding, and some institutions have problems with the purity of maize seeds owing to irregular management and operations during the breeding process. Precise and quantitative sowing methods have become mainstream, which has put forward new requirements for seed purity. Traditional detection methods include seed morphology identification, seedling identification, field planting, electrophoretic band identification, and molecular marker identification (Zhang et al., 2012; Ye et al., 2013; Rao et al., 2012). However, these methods have disadvantages, such as long identification times, high costs, and a destructive effect on seeds (Huang et al., 2014). Therefore, it is necessary to develop a non-destructive and rapid method for identifying maize seed varieties. Near-infrared spectroscopy and machine vision technologies have been widely applied in the field of agricultural product inspection (Gao et al., 2020). Near-infrared spectroscopy can more accurately detect the internal composition of a sample, such as protein and moisture (Serrano et al., 2021). Machine vision technology obtains characteristic information of a sample through image analysis. However near-infrared spectroscopy is based on the sampling method of rotating and diffuse reflection integrating spheres (Xie & Guo, 2020). Because the light spot can only be projected over a limited area, only part of the position information of the sample can be detected, which causes the spectral data to be less representative (Sun et al., 2021b). Therefore the detection results sometimes cannot achieve the desired effect. When the appearance characteristics of the samples are highly similar, machine vision technology cannot achieve a satisfactory result, because it can only reflect the physical appearance information from the samples, rather than the internal spatial and chemical information (Huang et al., 2022). Hyperspectral imaging (HSI) technology is a new nondestructive testing technology that combines spectral and image information (Zhang, Dai & Cheng, 2021). This technology has been developed to create an important seed purity detection method. HSI technology avoids the time-consuming processes involved early maize seed purity identification methods. These processes are long, expensive and destructive for seeds. HSI technology does not collect spectral information of a certain point, but obtains the spectral information of each pixel on the image (Huang et al., 2016). HSI technology combines the advantages of near-infrared spectroscopy technology and machine vision technology, so it can realize rapid, non-destructive, and efficient identification of the purity of maize seeds. Several studies have used HSI technology to identify the purity of seeds of different crops, and achieved good results, which verified the advantages of HSI technology (Aulia et al., 2022; Zhou et al., 2021; Bai et al., 2020; Zhou et al., 2020; Xia et al., 2019).

Ensemble learning is currently attracting considerable attention in the field of machine learning (Roshan & Asadi, 2021). It is usually better to identify seed purity based on ensemble learning technology than to use a single classifier (Karegowda, 2014). Wei et al. (2020) used random subspace ensemble learning to classify and identify different varieties of soybean seeds. Results showed that the variety identification model based on random subspace ensemble learning had high precision and stability. The using of random subspace ensemble learning to identify maize seed varieties has rarely been reported.

To improve the identification of maize seed varieties, SNV, MSC, and SG1 were used to pre-process the original spectral data. CARS and IRIV were used to extract characteristic bands of the full-band spectral data. KNN, SVM, LDA, and decision tree were used to establish maize seed varieties. IRIV has good feature extraction ability for high-dimensional data, and its use has not yet been reported for maize seed variety identification (Xu et al., 2021; Sun et al., 2018b; Yun et al., 2014; Sun et al., 2018a). Based on different pre-processing and characteristic band extraction methods, maize seed variety identification models based on the base learner are quite different. To improve the effectiveness of variety identification, an LDA-based random subspace ensemble learning maize variety identification model was used to achieve the rapid and non-destructive detection of maize seed varieties.

## Materials & Methods

### Materials

**Figure 1:**
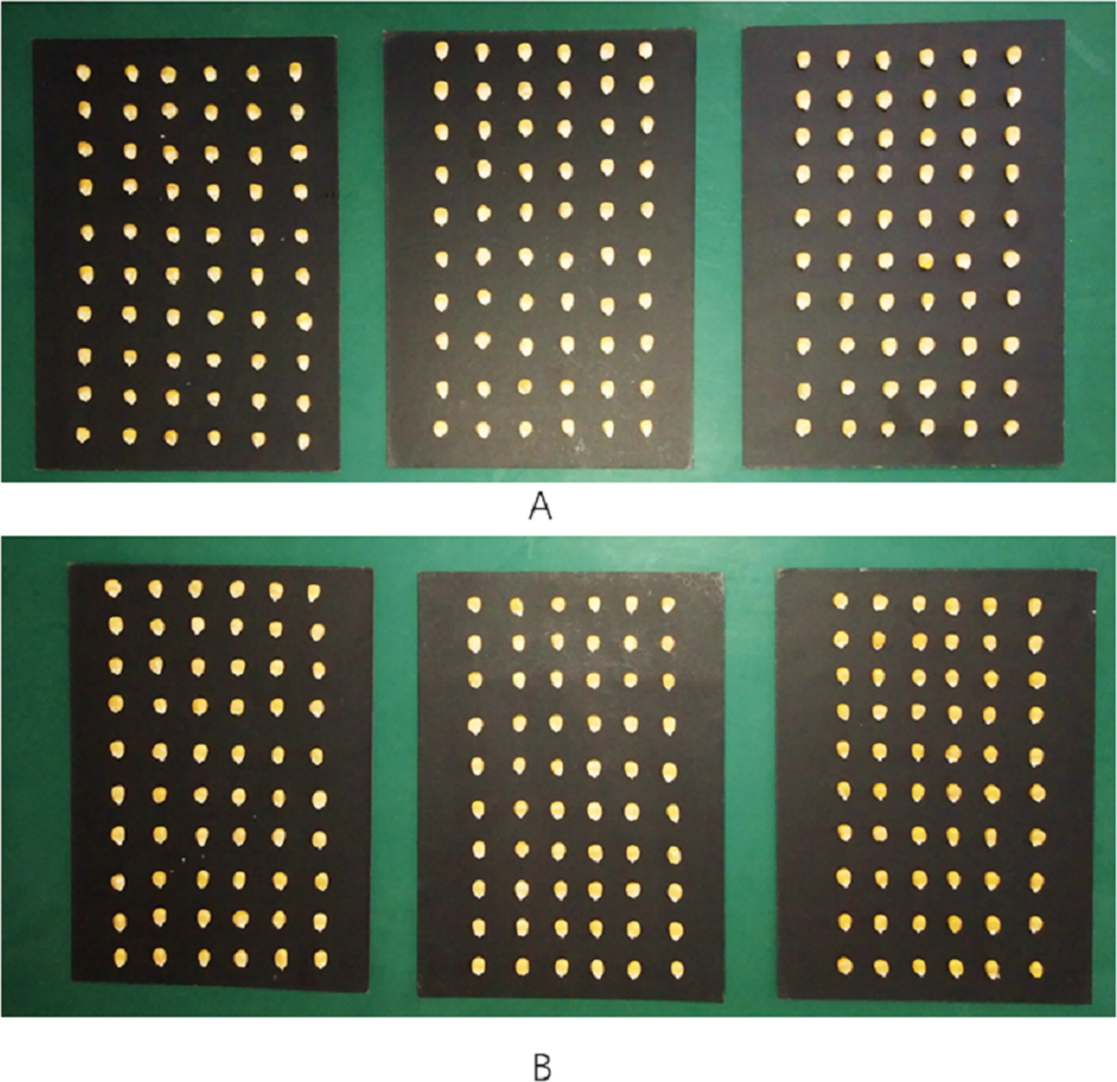
Six varieties of maize seeds. (A) Longping 206 Longping 208 Longping 259. (B) Huawan 263 Huawan 267 HuaWan 617.

The seeds used in the experiment were provided by Anhui Longping High-tech Seed Industry Co. Ltd (Anhui, China). There were 6 varieties used, namely ‘Longping 259’, ‘Longping 206’, ‘Longping 208’, ‘Huawan 263’, ‘Huawan 267’, And ‘Huawan 617’. There were 60 seeds of each variety used, comprising a total of 360 seeds. The maize seeds of each variety were of normal quality, and without any treatment or blemishes on the surface as shown in Fig. 1. Moreover, the morphological character and color of the six varieties of maize seeds were highly similar, and there was no significant difference between them. Before the experiment begins, all samples were stored in sealed bags and placed in a glass dryer. For the rigor and scientificity of the experiment, according to the prescribed standards of relevant sample collection, the purity of maize seeds involved in the experiment has reached more than 99%. According to the theory of probability and statistics, there will not be more than one hybrid sample in each variety of maize, so the samples of these six varieties of maize can be assumed as standard samples without hybrid among varieties. This study is carried out on this assumption.

### Hyperspectral acquisition system

**Figure 2:**
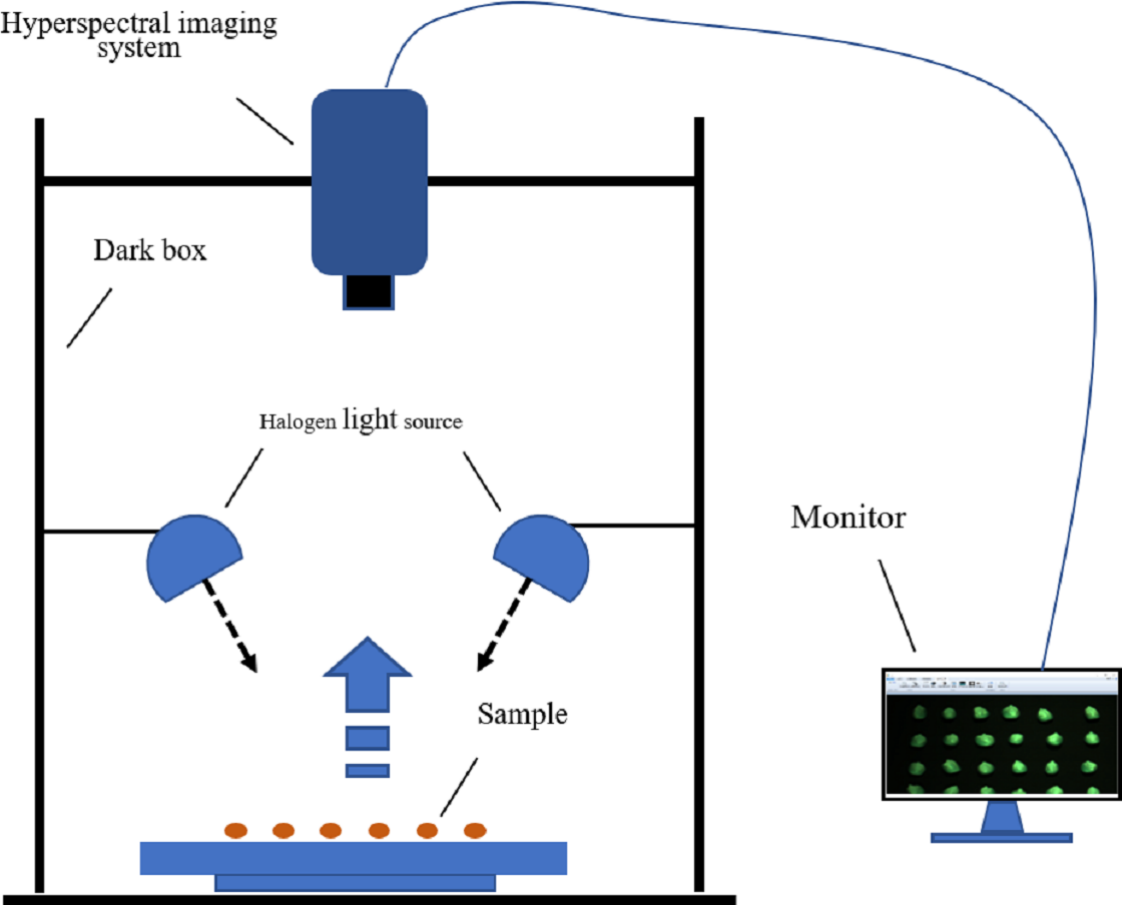
Hyperspectral imaging acquisition system.

The hyperspectral imaging system and its accessories constitute the hyperspectral data-acquisition system, as shown in Fig. 2. A GaiaField-Pro-V10 imaging spectroscopy system (JiangSu Dualix Spectral Imaging Technology Co. Ltd) was used to collect the spectral data from the maize seeds. The GaiaField-Pro uses a built-in push-broom mode for image acquisition. Therefore, no mobile platform is required. When collecting hyperspectral data, the area array detector and the imaging spectrometer are combined, and driven by the scanning control motor. The slit of the imaging spectrometer and the sample placement platform move relative to each other. The detector collects target information in real time, and finally stitches together to form a complete picture of the cube data. The spectral band range collected by this system is 400∼1000 nm. (visible-near-infrared band), with a total of 176 bands. The exposure time is set to 0.6 ms, the focal length is 30.51 cm, and the resolution is 3.2 nm. The width of the spectrometer slit is 30 µm, and the length is 9.6 nm. The scanning speed was 15 s/cube; the detector calibration method and spectral calibration were used, and the numerical aperture was F/2.8.

### Data collection and black and white correction

Before the experiment began, the halogen light source of the hyperspectral data acquisition system was preheated for 10 min to ensure the stability of the light source. Because the GaiaField-Pro-V10 hyperspectral imaging system integrates a data acquisition and analysis processing system, no external computer is required, and only a display is connected outside the system to display the data acquisition process in real time. Maize seeds of each variety were placed on a black panel with low reflectivity in six rows and ten columns. The black panel can isolate the background, except the maize seeds. This can eliminate the influence of environmental interference information on the hyperspectral data to a certain extent, so that only the maize seeds were in the spectral field of view (Sun et al., 2021a). A total of 360 maize seeds were collected to pick up data, and 60 seeds were collected each time. the varieties of maize seeds collected each time were the same, so a total of 6 data collections were conducted. During the experiment, to reduce the influence of dark current and uneven illumination of the hyperspectral acquisition system, black-and-white correction was performed on the hyperspectral image (Zhang, Rao & Ji, 2020). The standard whiteboards were made of Teflon. The correction formula is given by equation:

\begin{eqnarray*}R= \frac{{I}_{raw}-{I}_{dark}}{{I}_{white}-{I}_{dark}} \end{eqnarray*}

where *I*_*raw*_ is the raw data of maize seed hyperspectral imaging, *I*_*dark*_ is the dark current data (the reflectance is close to 0), *I*_*white*_ is the whiteboard data (the reflectance is close to 1), and R is the final hyperspectral imaging of maize seeds after black and white correction. The black and white correction tool was the built-in calibration software of the GaiaField-Pro-V10 imaging spectroscopy system.

### Extraction of the region of interest

The data collected by the HSI system contains the image information and spatial data information of the maize seeds.

Therefore, the original data must be processed to extract the spectral data. The entire area of a single maize seed in the image is taken as the region of interest (ROI), and then the average reflectance of all pixels in the ROI is calculated as the spectral value of each maize seed (Qi et al., 2017). Its value can be calculated using the following equation:

\begin{eqnarray*}A= \frac{\sum _{i=1}^{176}\sum _{j=1}^{m}{A}_{ij}}{m} \end{eqnarray*}

*m*, which is the number of all pixels in the ROI area, and *A*_*ij*_ is the spectral value of the i-th pixel in the j-th band.

**Figure 3:**
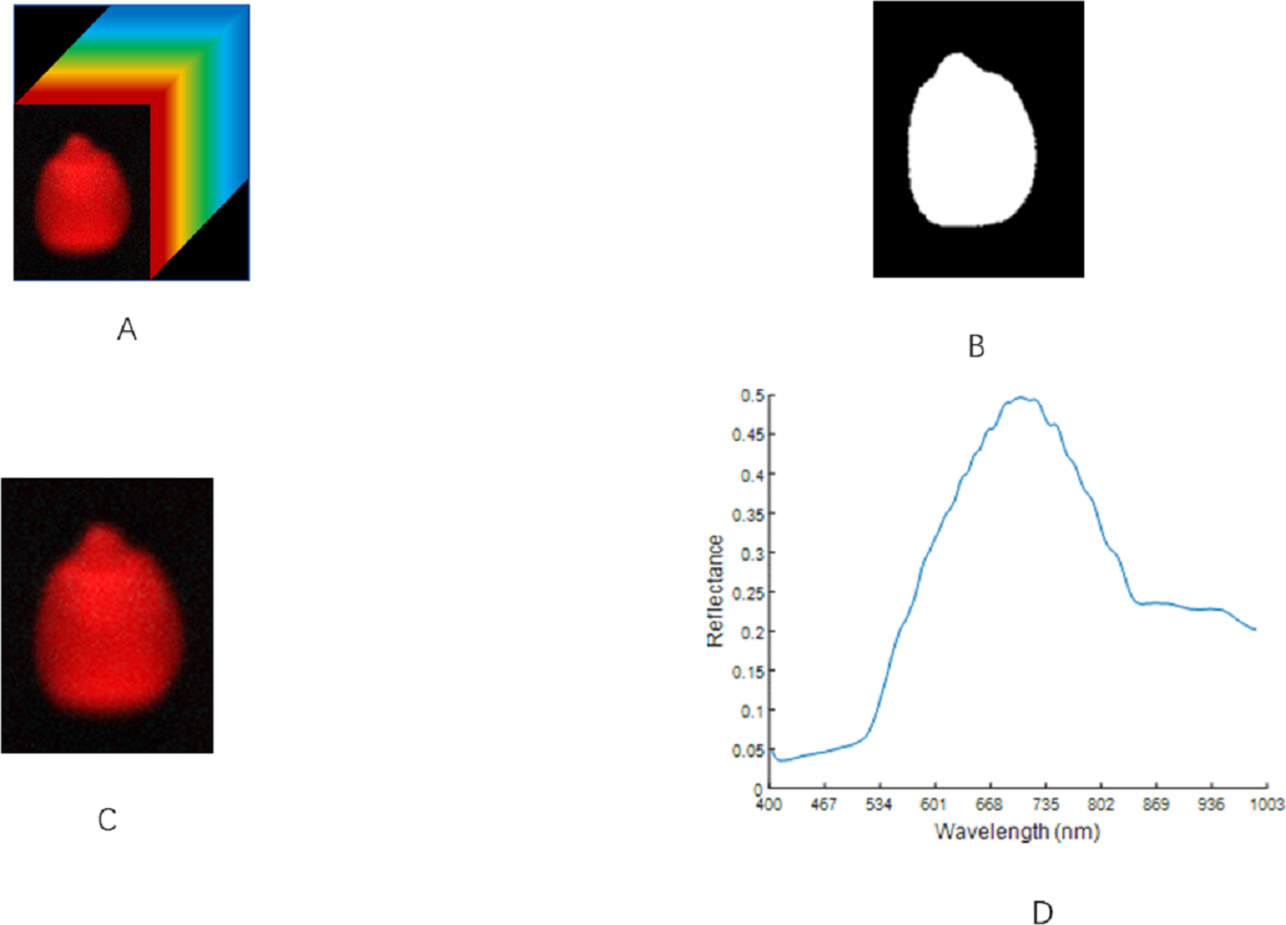
ROI extraction steps of maize seeds. (A) Hyperspectral image. (B) Binarized image. (C) Region of interest. (D) The original spectrum.

The threshold method was used to remove the background region (Thakur & Madaan, 2014). In line with the difference in the gray value between the ROI and the background area, a threshold was set for the gray image. The pixel with a gray value greater than the threshold was set to 1. Otherwise, it was set to 0, and the gray image was converted into a binarized image. The binarized image was then applied to the original image to separate the maize seed hyperspectral image from the background area. The selection of the ROI area was completed using MATLAB R2019b software. After many experiments, when the threshold was set to 0.1, the background area could be effectively separated with the condition that the integrity of the ROI could be guaranteed. The spectral average of all the pixels in the image was taken as the spectral data for the region. This process is illustrated in Fig. 3.

### Preprocessing of spectral data

MSC, SNV, and SG1were used to preprocess the original spectral data. Eliminating interference signals such as background noise, baseline drift, and stray light during spectral acquisition reduced the complexity and improved the interpretability of the model (Wang et al., 2018). After optimizing the selection of parameters, the order of derivation in the SG1 algorithm was set to 1, the number of window points was set to 5, and the degree of the polynomial was set to 2.

### Modeling based on base classifiers

CARS and IRIV were used to extract characteristic wavelengths to reduce computational consumption and increase computational speed, eliminate irrelevant or nonlinear variables, and obtain a model with strong predictive ability and good robustness (Lu et al., 2018).

IRIV is a method of selecting feature variables based on a binary matrix shuffling filter (BMSF) (Liang et al., 2014). According to the degree of contribution to the model, the variables were divided into strong information variables, weak information variables, no information variables, and interference information variables. After many iterations, the non-informative variables and interfering information variables were removed. The strong and weak information variables were retained, the weak information variables were eliminated in reverse, and the remaining strong information variables were used as feature variables (Song et al., 2019).

CARS combines Monte Carlo sampling with a partial least-squares regression algorithm, and is a common feature wavelength selection method (Jiang et al., 2021). The characteristic wavelength extraction process is as follows: (1) the Monte Carlo sampling method is first used to select the correction set. (2) A partial least squares (PLS) model is established based on the selected correction set. (3) The absolute value of the regression coefficient is calculated, the variables with large absolute values are retained, and the exponential decay function is used to determine the number of variables to be eliminated. (4) A PLS cross-validation model is established, and the subset corresponding to the PLS model with the smallest cross-validation root mean square error is selected, which is the required characteristic wavelength (Huan et al., 2021).

In IRIV, the maximum number of principal components was set to 40, and the number of cross-validations was 11. In CARS, after MSC preprocessing of the original spectrum data in CARS, the maximum number of principal components was set to 25. Then, a 10-fold cross-validation was used to establish the PLS model, with a Monte Carlo sampling frequency of 80.

The selected classifiers were decision tree (DT), line discriminant analysis (LDA), support vector machines (SVM), and k-nearest neighbor (KNN). In DT, the maximum number of splits is set to 20, and the splitting criterion is the Gini diversity index. In SVM the kernel function is linear, the frame constraint is set to 3, and the kernel scale is set to automatic. In KNN, the number of neighbors is set to 20, the distance measure is Minkowski, and the distance weight is equal to the distance.

The KS method (Kennard–Stone) was used to divide the sample set into training and prediction sets. The Euclidean distance formula was used to calculate the distance between each two samples, before dividing he two samples with the longest distance into the training set. The distances were then calculated between the remaining samples and the two samples that were selected. The distances are the smallest and the largest sample is included in the training set. This step is then repeated until the number of training set samples reaches a predetermined value (Luo et al., 2021). The distance calculation formula is shown in the following equation (Li et al., 2014).

\begin{eqnarray*}{d}_{x} \left( p,q \right) =\sqrt{\sum _{j=1}^{N}[{x}_{p} \left( j \right) -{x}_{q} \left( j \right) ]^{2}} \end{eqnarray*}

where *d*_*x*_*(p,q)* is the Euclidean distance between two samples *p* and *q*, *p,q* ∈[1,*N* ].

### Modeling based on random subspace ensemble learning

**Figure 4:**
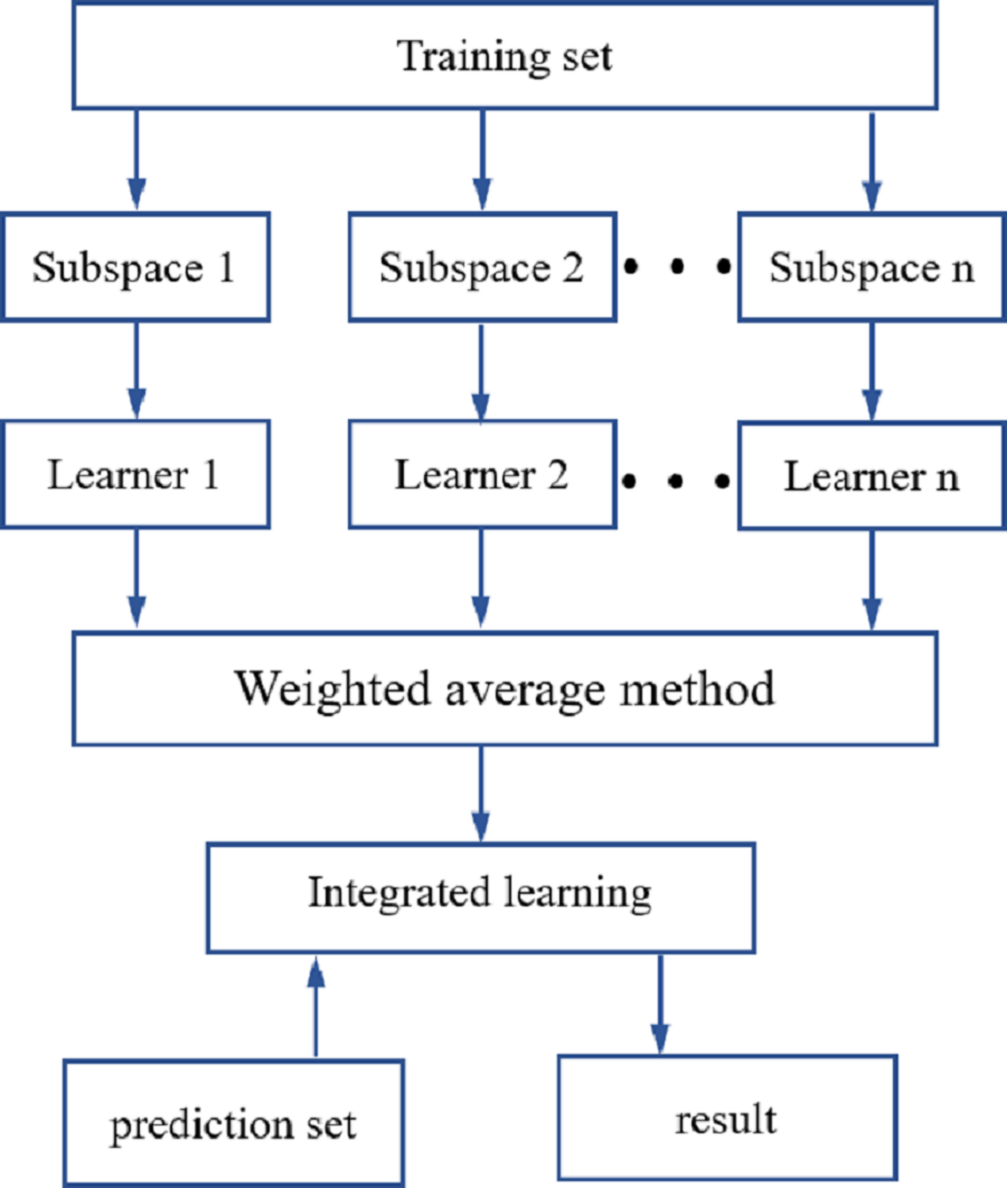
Random subspace ensemble learning flow chart.

ROI processing was performed on the hyperspectral images of the maize seeds, and the hyperspectral data for each maize seed were then extracted. The original spectral data were preprocessed using SNV, SG1and MSC. The spectral characteristic wavelengths were extracted using the CARS and IRIV methods. Using LDA, DT, SVM and KNN-based classifiers, an identification model for the different maize seed varieties was established. To further improve the precision in identifying different maize seed varieties, an integrated learning method was used that was based on a random subspace to improve the robustness and generalization ability of the maize seed identification model. The flow of the random subspace integration method is illustrated in Fig. 4.

The random subspace ensemble learning used in this study was used to generate N training subsets with the same number of samples in the training set. These are low-dimensional feature subspaces, but the dimensionality was lower than that of the training set data. The data from the N low-dimensional data subspaces were inputted into the base learner for training (Zhou, 2012; Zhang & Ma, 2012). In line with the performance of the base learner, it was decided whether to use the base classifier, which was the combination strategy of the base learner. The combined strategy used in this study was the weighted average method. The random subspace reduces correlation of each base learner using a random subset of features instead of all the features used to train each base learner (Ho, 1998).

## Results and Discussion

### Spectral acquisition and raw spectral analysis

**Figure 5:**
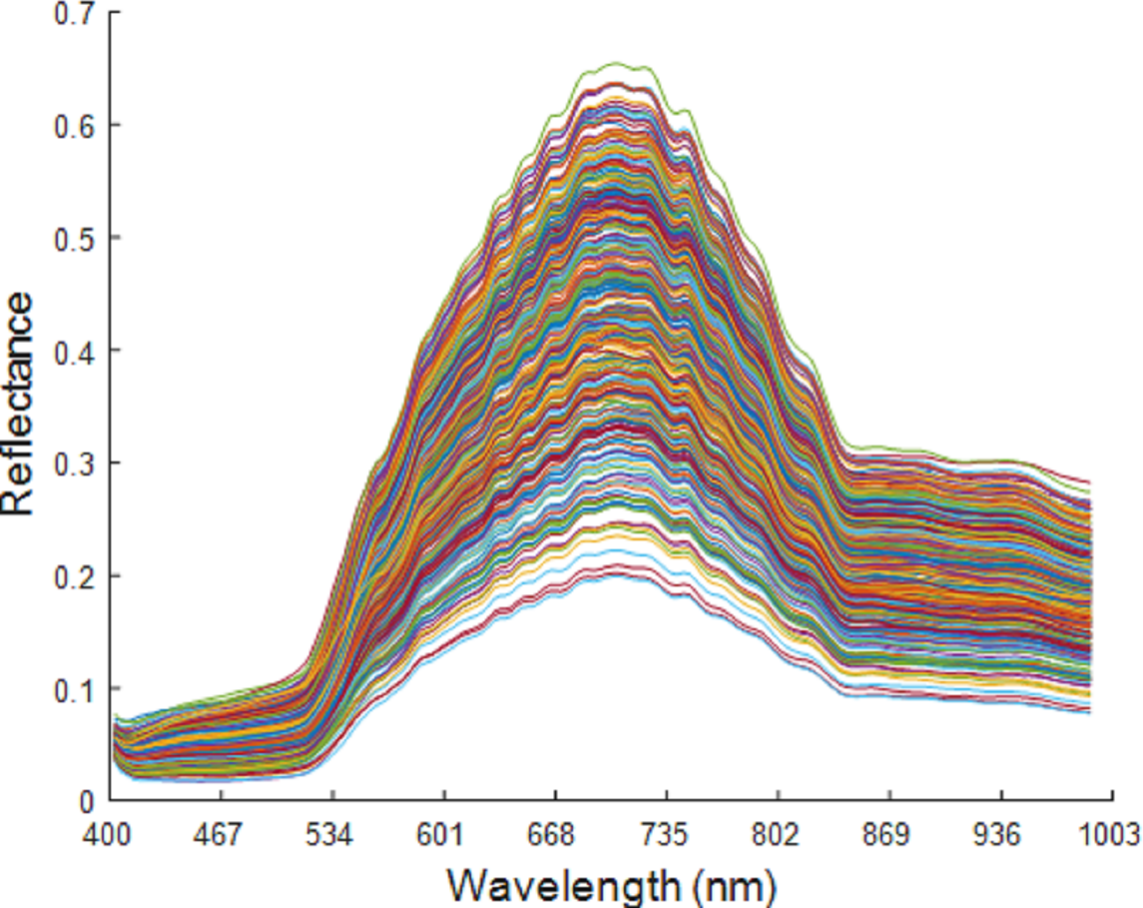
Original spectrum of maize seeds of six varieties.

The spectral band ranged from 400 to 1,000 nm, with a total of 176 wavelengths. At both ends of the spectral wavelength range, the spectral curve was flat and with no obvious fluctuation. This indicates that the influence of the interference information in the system and in the environment on the spectral data is negligible, which therefore preserves all the wavelengths. The original spectral curve is shown in Fig. 5.

**Figure 6:**
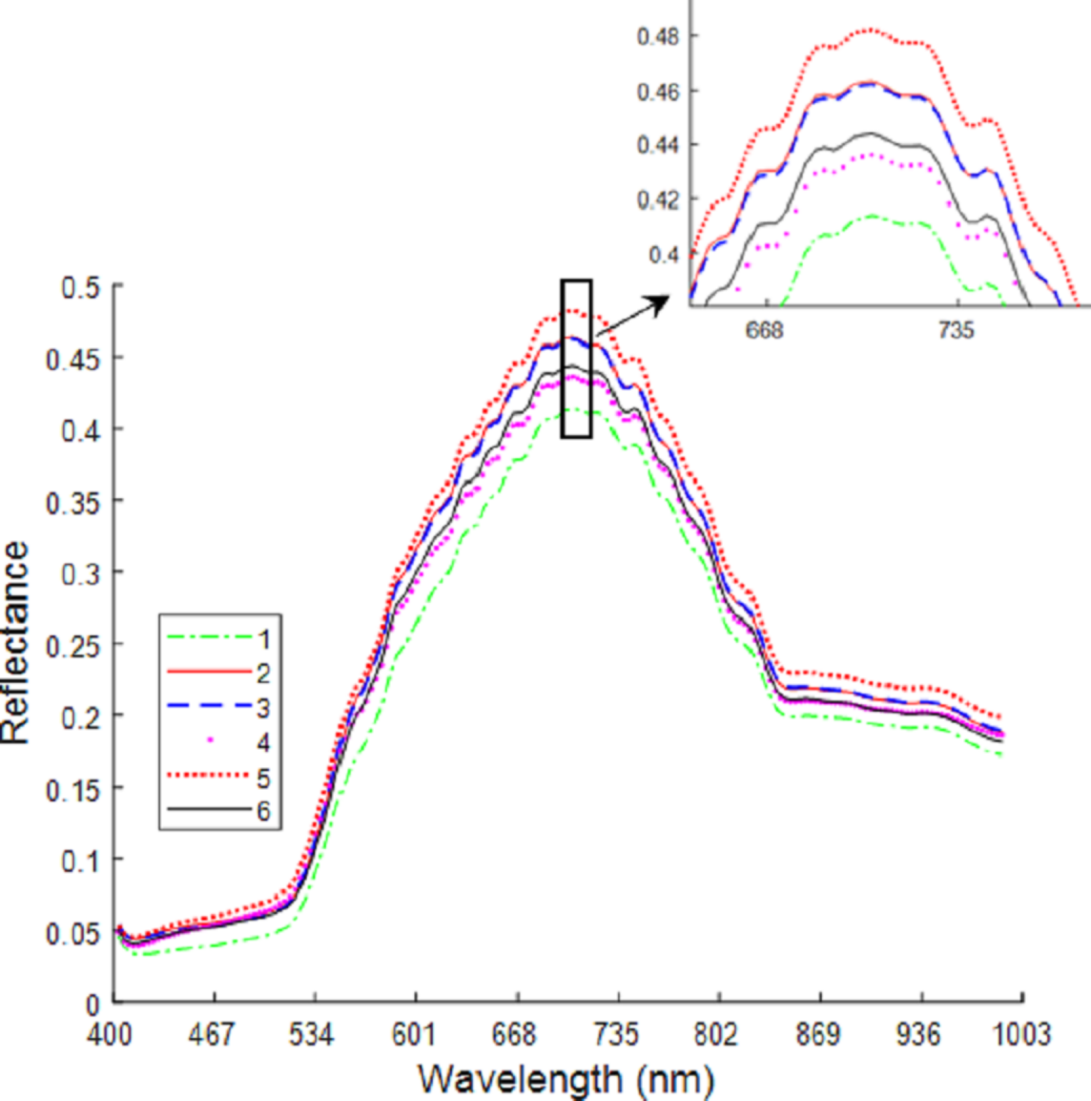
Average spectrum of maize seeds from six varieties.

Figure 6 shows the average spectral curves for the six maize seed varieties. The trend in the average spectral curve for the six maize seed varieties shown in Figs. 3–2 is predominantly consistent. It is difficult to identify the maize varieties on the trend of the curve, but in the wavelength range of 668–735 nm, the six maize varieties can be differentiated. The distance between the spectral curves reaches the maximum value, so the difference in spectral reflectance for the six maize seed varieties is the most pronounced. The order from high to low for the spectral reflectance is “Huawan 267” (code-named 5), “Longping 206” (code-named 2), “Huawan 263” (code-named 3), “Longping 208” (code-named 6), “Huawan 617” (code-named 4), “Longping 259” (code-named 1), which may be caused by the different contents of protein, starch, oil and water in them. For maize seeds, the spectral reflectance at 410–500 nm is proportional to the protein content, and the presence of starch, oil and other compounds leads to the absorption peak at 500–735 nm. The peak near 980nm shows the central absorption wavelength of the second overtone of O-H stretching, which is caused by the presence of water and carbohydrates, or by the second overtone of OAH stretching due to the interaction between water and protein. The spectral curves for the maize varieties code-named “2” and “3” are the closest, with the longitudinal distance between the two spectral curves being the smallest. It can, therefore, be inferred that these two varieties are similar. If the seeds of these two varieties are mixed together, it will likely be difficult to distinguish them with the naked eye. Different maize seed varieties have varying seed vigor, germination vigor, disease resistance, and lodging resistance. If the two maize seed varieties are mixed together, sowing may lead to uneven seedling emergence. Dwarf seedlings will have insufficient photosynthesis due to lack of light, ultimately affecting the crop harvest. In addition, in the spectral curves of the maize seeds code-named “6” and “4”, the vertical distance between them is also relatively close. This may also bring certain difficulties in identifying specific maize of varieties. Therefore, the identification of maize varieties with the techniques developed in this study is important and plays a key role in promoting the development of maize seed identification.

### Spectral data preprocessing and feature wavelength extraction

**Figure 7:**
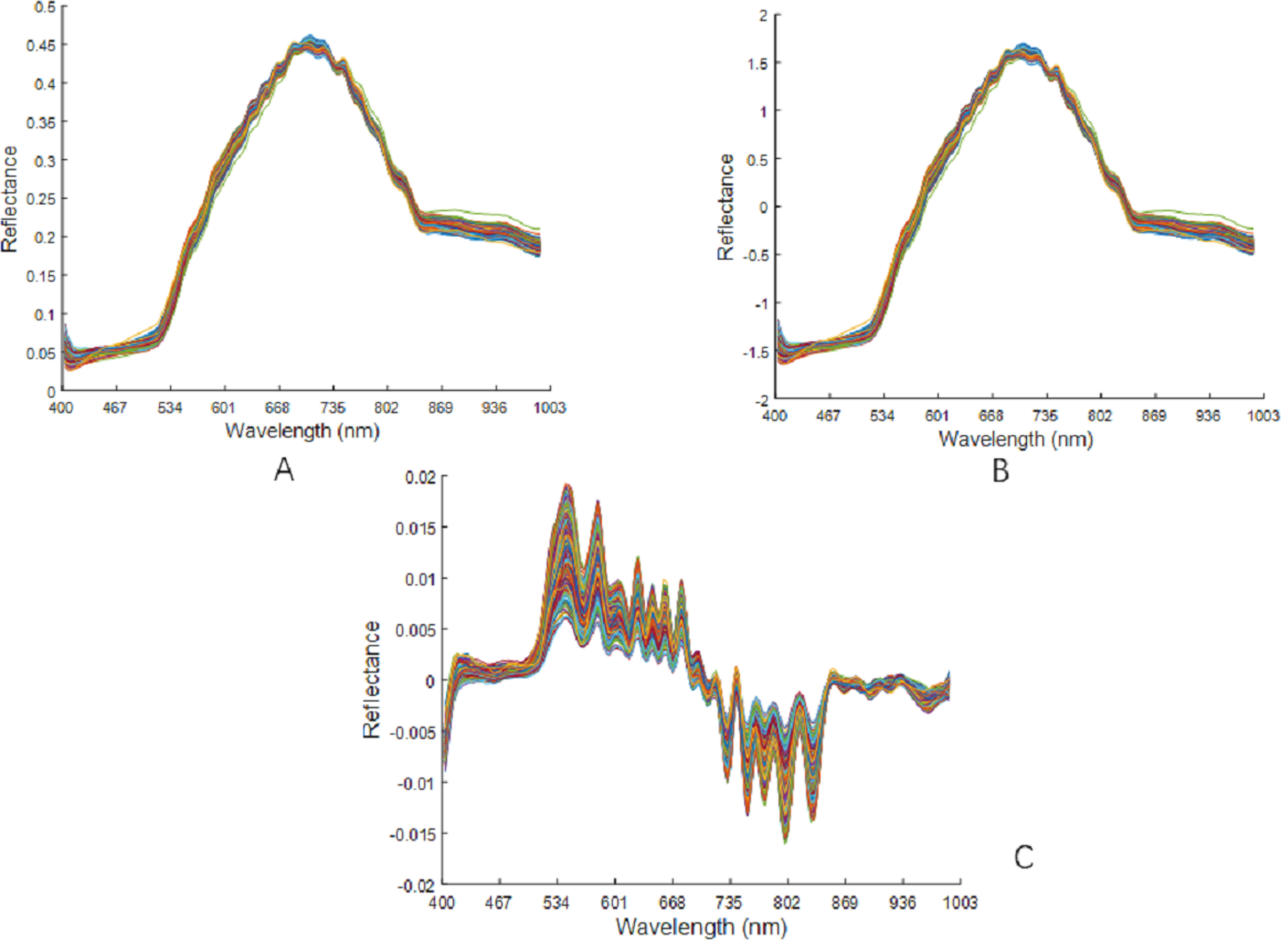
Preprocessing results of raw spectral data. (A) Spectra after MSC pretreatment. (B) Spectra after SNV pretreatment. (C) Spectra after SG1 pretreatment.

The raw spectral data were preprocessed using MSC, SNV, and SG1, as shown in Fig. 7. There was no changes in the peak position of the curve after the MSC pretreatment. The spectral curve from after the SNV pretreatment was very similar to the position of the curve after the MSC pretreatment. After the SG1 pre-treatment, there was a significant increase in the absorption peaks of the spectral curves.

Feature variable extraction was performed on the pre-processed spectral data using CARS and IRIV. In IRIV, the maximum principal component was set to 40 and the number of cross-validation was 11; In CARS, the maximum principal component was 25, the number of Monte Carlo samples is 80, and the PLS model is established by 10-fold cross-validation method. When CARS was used to extract features from the preprocessed data from the MSC, SNV, and SG1, the number of selected feature variables were 71, 60, and 71, respectively. When using IRIV to extract features from the preprocessed data from the MSC, SNV, and SG1, the numbers of the selected feature variables were 69, 73 and 64, respectively.

### Maize variety identification by base classifier

After denoising and dimensionality reduction of the original spectral data from the maize seeds using three preprocessing methods and two feature variable extraction methods, namely decision tree, KNN, SVM and LDA, variety identification models were established. The classification accuracies of the training and test sets are listed in Table 1. Before spectral data feature extraction had been performed, classification modeling precision based on the decision tree was the lowest, and the classification precision based on the LDA was the highest. After feature variable extraction, the classification precision of the LDA significantly improved. The accuracies of the training and prediction sets were 0.9626 and 0.9333, respectively. Based on the SVM modeling, the degree of change in the precision of variety identification was less pronounced both before and after feature extraction. The classification precision based on KNN and DT was lower than 0.55 before and after the feature variable extraction. This indicates that using a single classifier to model maize seed varieties is prone to low levels of precision. Compared with other preprocessing methods, the MSC-based variety identification model has a higher precision rate. Therefore, for subsequent variety identifications, the MSC method was used to improve the prediction precision. The identification of maize varieties based on the base classifier and the identification of maize seed varieties based on random subspace ensemble learning in this study were both looped 10 times in MATLAB R2019b. The average value was taken at the end to reduce the random error of the experimental results (Jang et al., 2022).

**Table 1:** Identification results of maize varieties based on the base classifier.

Dimensionality reduction method		NONE		CARS		IRIV	
Classifier	Preprocessing	Training set	Prediction set	Training set	prediction set	Training set	Prediction set
DT	MSC	0.4948	0.4667	0.5122	0.4333	0.5296	0.4111
	SNV	0.5156	0.4667	0.4944	0.4222	0.5226	0.4222
	SG1	0.3904	0.3444	0.3337	0.4111	0.3893	0.3667
KNN	MSC	0.5563	0.4556	0.5256	0.4222	0.5326	0.44440
	SNV	0.5378	0.4667	0.5148	0.4444	0.5389	0.4333
	SG1	0.4196	0.3444	0.4011	0.3444	0.3652	0.3222
SVM	MSC	0.7730	0.7333	0.7769	0.7444	0.766	0.7222
	SNV	0.7570	0.7444	0.7567	0.7222	0.7578	0.7333
	SG1	0.7378	0.6222	0.7093	0.6333	0.7159	0.6444
LDA	MSC	0.7881	0.8667	0.9617	0.9111	0.9626	0.9333
	SNV	0.7859	0.8667	0.9530	0.9111	0.9282	0.9333
	SG1	0.7133	0.8111	0.9341	0.9111	0.8889	0.9333

### Maize variety discrimination based on ensemble learning in random subspace

When using random subspace ensemble learning the first step is to determine the ensemble scale, that is, the feature dimension of the constructed subspace and the number of base classifiers. The combination of an appropriate number of feature dimensions and the number of base classifiers can improve the prediction efficiency while ensuring the prediction effect. When the number of base classifiers is determined, different feature dimensions have different levels of precision in identifying maize seed varieties (Ji et al., 2011). When modeling the full-band spectral data based on random subspace ensemble learning, 15, 20, 25, 30, 35, and 40 groups of six base classifiers were selected to explore the range of different feature dimensions in the range of 20∼100. The identification results are presented in Table 2. Table 2 shows that when the number of base classifiers is constant, the precision of variety identification increases with the subspace feature dimension. From the longitudinal observation of the table, when the subspace dimension is constant, there is no clear linear relationship between the precision of breed identification and the increase in the number of classifiers. However, there is an optimal combination of the number of base classifiers and the subspace dimension. When the subspace dimension increased to 80∼90, the precision of the variety identification had a downward trend. The combination of the number of base classifiers and the dimension of the subspace has a certain influence on the identification precision. Therefore, it is not the case that the greater the dimension of the subspace and the number of base learners, the higher the precision of breed identification, and the number of the two should be adjusted. This means that the variety identification effect of the random subspace can reach the best state. By adjusting the number of base classifiers and the dimension of the subspace features many times, the number of base classifiers was finally determined to be 25, with the dimension of the subspace being 63. Based on these two parameters, the maize seed varieties were identified. The precision of the training set was 0.9726, and the precision of the prediction set was 0.9467.

**Table 2:** The discrimination precision of the base classifier and the subspace dimension on the quality of maize seeds.

Number of base classifiers	Subspace dimension								
	20	30	40	50	60	70	80	90	100
15	0.8467	0.8989	0.9211	0.9333	0.9444	0.9422	0.9456	0.9400	0.9378
20	0.8500	0.8956	0.9200	0.9378	0.9456	0.9456	0.9467	0.9322	0.9367
25	0.8489	0.9044	0.9178	0.9400	0.9411	0.9422	0.9378	0.9400	0.9367
30	0.8478	0.8967	0.9200	0.9411	0.9444	0.9433	0.9400	0.9300	0.9367
35	0.8544	0.8944	0.9222	0.9378	0.9422	0.9456	0.9400	0.9322	0.9378
40	0.8567	0.9000	0.9167	0.9378	0.9422	0.9444	0.9389	0.9356	0.9378

After the preprocessed spectral data are modeled by the random subspace ensemble learning model of maize varieties, the number of basic learners and the subspace dimension of the random subspace ensemble learning are required to be re-determined because the dimension of the spectral data after feature extraction is reduced to 71. The number of learners was determined as six groups of 15, 20, 25, 30, 35, and 40, and the subspace dimension was adjusted at intervals of five within the range of 20–65, as shown in Table 3. Similar to the trend of the precision rate change based on full-band modeling, when the number of basic learners is constant, the precision rate of breed identification increases with an increase in the subspace dimension rate slightly. After several parameter selections, the number of base learners was 19, and the subspace dimension was 53. Based on these two parameters, the training set precision of the random subspace ensemble learning was 0.9644 and the prediction set precision was 0.9222. based on the same method of selecting the number of basic learners and the dimension of the subspace for the spectral data processed by CARS dimensionality reduction, two parameters were selected for the spectral data preprocessed by IRIV, and the precision of maize seed variety identification was obtained. The highest precision rate was 0.9556, which was 3.23% higher than that of the CARS feature extraction method, as shown in Table 4. Finally, it was determined that the number of base classifiers was 33 and the dimension of the subspace features was 53. The training set precision rate of the random subspace ensemble learning based on these two parameters was 0.9644, and the prediction set precision rate was 0.9556.

**Table 3:** Identification precision of maize seed varieties based on base classifier and subspace dimension after CARS.

Number of base classifiers	Subspace dimension									
	20	25	30	35	40	45	50	55	60	65
15	0.8256	0.8778	0.9044	0.9033	0.9078	0.9144	0.9222	0.9167	0.9189	0.9122
20	0.8489	0.8778	0.9022	0.9078	0.9111	0.9178	0.9200	0.9133	0.9144	0.9122
25	0.8356	0.8756	0.9000	0.9033	0.9033	0.9156	0.9211	0.9167	0.9122	0.9111
30	0.8478	0.8767	0.9022	0.9100	0.9089	0.9167	0.9189	0.9156	0.9111	0.9111
35	0.8500	0.8882	0.9044	0.8989	0.9044	0.9122	0.9189	0.9122	0.9178	0.9122
40	0.8433	0.8833	0.9067	0.9022	0.9033	0.9133	0.9200	0.9178	0.9133	0.9111

**Table 4:** Identification precision of maize seed varieties based on base classifier and subspace dimension after IRIV.

Number of base classifiers	Subspace dimension									
	20	25	30	35	40	45	50	55	60	65
15	0.8678	0.8944	0.9144	0.9300	0.9411	0.9511	0.9544	0.9556	0.9489	0.9367
20	0.8767	0.8978	0.9100	0.9233	0.9444	0.9522	0.9556	0.9533	0.9533	0.9389
25	0.8882	0.8989	0.9122	0.9278	0.9411	0.9533	0.9556	0.9522	0.9500	0.9400
30	0.8800	0.8956	0.9111	0.9244	0.9400	0.9489	0.9556	0.9556	0.9533	0.9411
35	0.8789	0.8978	0.9100	0.9233	0.9433	0.9533	0.9544	0.9556	0.9533	0.9344
40	0.8767	0.8989	0.9111	0.9233	0.9411	0.9522	0.9556	0.9556	0.9544	0.9389

**Figure 8:**
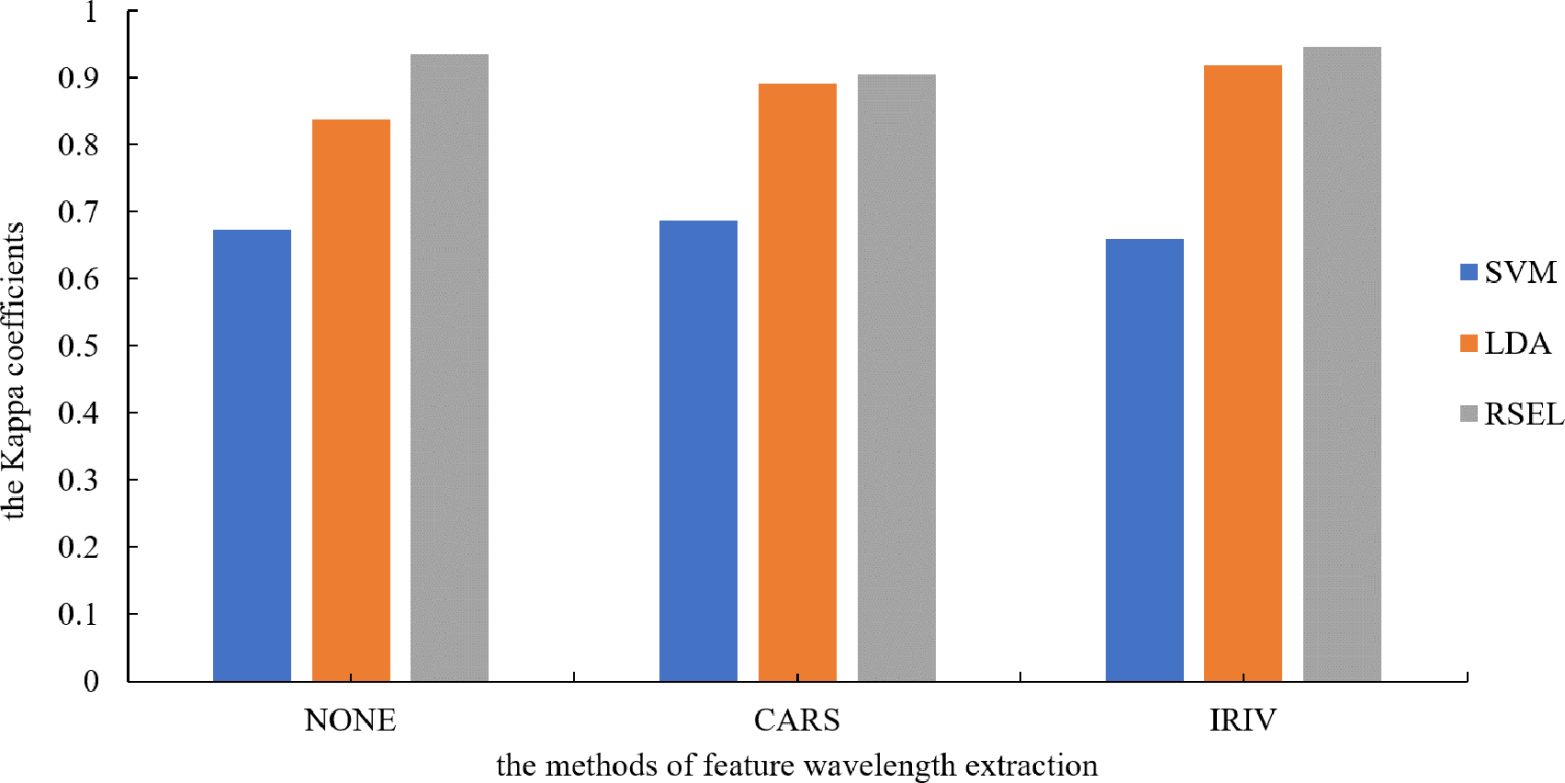
Kappa coefficients for different modeling methods.

**Figure 9:**
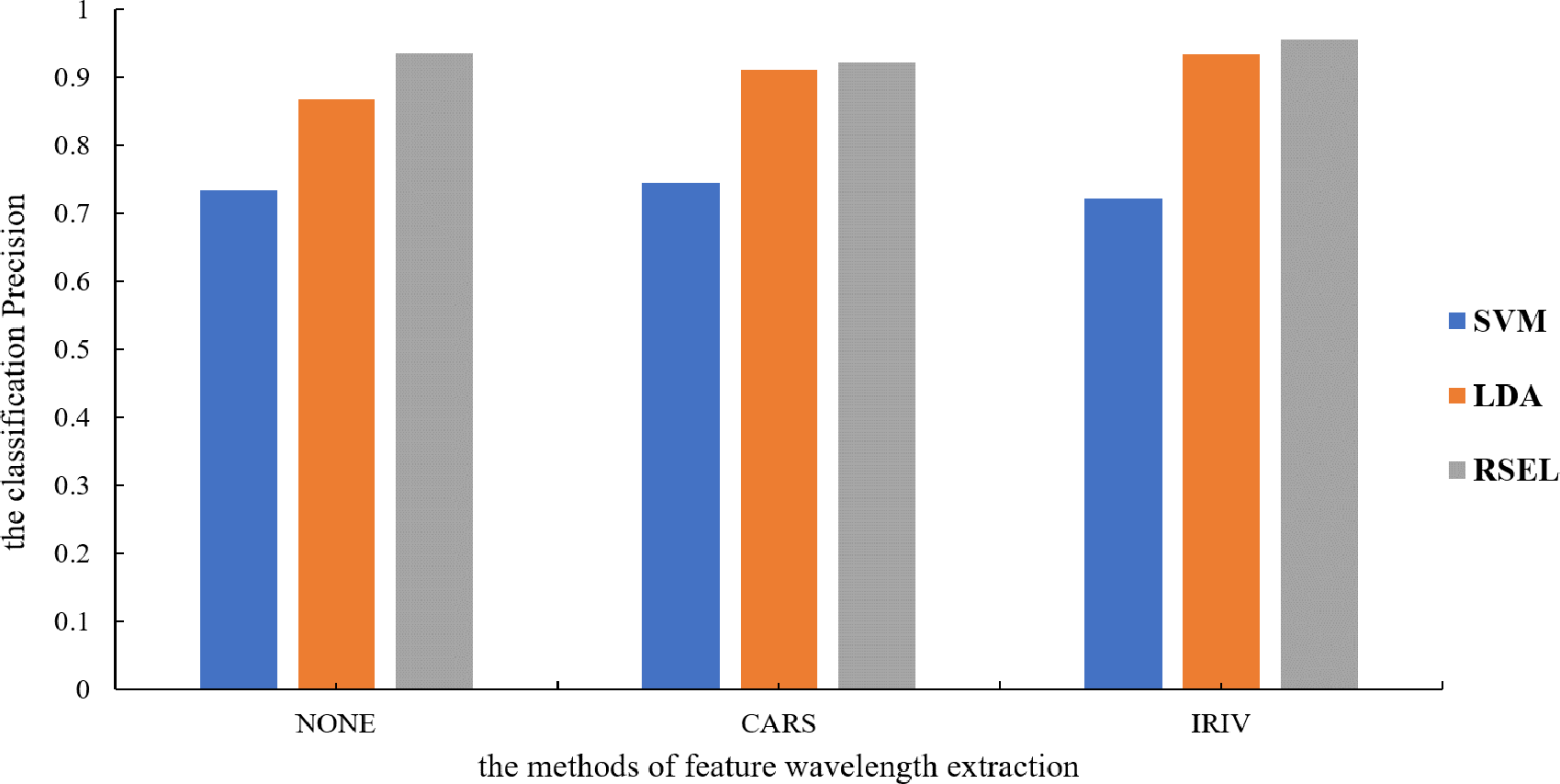
Modeling precision of different modeling methods.

It can be seen from Table 1 that the SVM and LDA have a higher recognition precision of maize seed varieties by the base classifier. Therefore, the results for varietal recognition of maize seeds using random subspace ensemble learning were compared with those of the SVM and the LDA. The two parameters that were compared were breed identification precision and the Kappa coefficient. The Kappa coefficient is a measure of classification precision based on the confusion matrix. It typically evaluates a value between 0 and 1. Figures 8 and 9 show that the discrimination results based on the SVM are not significantly different both before and after the dimension reduction of the spectral data. Based on the identification results of the LDA, after the spectral data dimensionality reduction, the precision and the Kappa coefficient significantly improved. For the classification results for spatial ensemble learning, after IRIV dimensionality reduction processing, the discrimination precision and Kappa coefficient improved. The two parameters decreased slightly after CARS processing. Overall, the use of random subspace ensemble learning for cultivar identification for six maize seed varieties had higher precision and Kappa coefficient than the base classifier.

## Conclusion

Using hyperspectral imaging technology combined with random subspace ensemble learning, classification and identification were undertaken for six maize seed varieties. The hyperspectral image of the endosperm side of maize seeds was collected, the ROI area of the maize seeds was extracted using the threshold method in MATLAB software, and the average spectral information of the seeds was extracted. MSC, SNV and SG1 were used to preprocess the original spectral data, and then the characteristic wavelengths were extracted using CARS and IRIV. The classification effects of DT, LDA, SVM and KNN were compared, the MSC preprocessing method and the LDA method were selected. A base classifier was used for ensemble learning with LDA as a random subspace. Results show that this model based on maize-MSC-IRIV-RSEL can improve the classification precision of the base learner from 0.9333 to 0.9556, and the Kappa coefficient from 0.9174 to 0.9457. Results show that the use of hyperspectral imaging technology combined with stochastic subspace ensemble learning can further improve the classification of maize seed varieties and achieve rapid non-destructive detection of maize seed purity.

The subspace ensemble learning algorithm used in this study can fuse multiple base classifiers into a strong classifier, which can enhance the precision and stability of the maize seed purity identification model. However, there are some areas that need to be improved. Firstly, not all subspace data can contribute to the improvement of the final results. Subsequent researchers can develop more efficient algorithms to find subspaces that can improve the results. Secondly, the base learner used in this study is LDA, which has achieved good results. Subsequent researchers can find better base classifiers to improve the recognition effect of maize seed purity.

##  Supplemental Information

10.7717/peerj-cs.1354/supp-1 Supplemental Information 1 The hyperspectral data information of maize seeds with the variety ‘huawan617’ Each row represents the hyperspectral data information of a seed. There are 60 rows representing the data information of 60 seeds. Each column represents the band information of each seed, and there are 176 columns in the table, representing 176 bands.

10.7717/peerj-cs.1354/supp-2 Supplemental Information 2 The hyperspectral data information of maize seeds with the variety ‘longping208’ Each row represents the hyperspectral data information of a seed. There are 60 rows representing the data information of 60 seeds. Each column represents the band information of each seed, and there are 176 columns in the table, representing 176 bands.

10.7717/peerj-cs.1354/supp-3 Supplemental Information 3 The hyperspectral data information of maize seeds with the variety ‘longping206’ Each row represents the hyperspectral data information of a seed. There are 60 rows representing the data information of 60 seeds. Each column represents the band information of each seed, and there are 176 columns in the table, representing 176 bands.

10.7717/peerj-cs.1354/supp-4 Supplemental Information 4 The hyperspectral data information of maize seeds with the variety ‘longping259’ Each row represents the hyperspectral data information of a seed. There are 60 rows representing the data information of 60 seeds. Each column represents the band information of each seed, and there are 176 columns in the table, representing 176 bands.

10.7717/peerj-cs.1354/supp-5 Supplemental Information 5 The hyperspectral data information of maize seeds with the variety ‘huawan263’ Each row represents the hyperspectral data information of a seed. There are 60 rows representing the data information of 60 seeds. Each column represents the band information of each seed, and there are 176 columns in the table, representing 176 bands.

10.7717/peerj-cs.1354/supp-6 Supplemental Information 6 The hyperspectral data information of maize seeds with the variety ‘huawan267’ Each row represents the hyperspectral data information of a seed. There are 60 rows representing the data information of 60 seeds. Each column represents the band information of each seed, and there are 176 columns in the table, representing 176 bands.

10.7717/peerj-cs.1354/supp-7 Supplemental Information 7 Codes

## Additional Information and Declarations

### Data Availability

The following information was supplied regarding data availability: The raw data and code is available in the Supplemental Files.
